# The Mechanisms of Molybdate Distribution and Homeostasis with Special Focus on the Model Plant *Arabidopsis thaliana*

**DOI:** 10.3390/molecules29010040

**Published:** 2023-12-20

**Authors:** Jan-Niklas Weber, Rieke Minner-Meinen, David Kaufholdt

**Affiliations:** Institut für Pflanzenbiologie, Technische Universität Braunschweig, Humboldtstrasse 1, D-38106 Braunschweig, Germany

**Keywords:** molybdate transporter, molybdate distribution, homeostasis, *Arabidopsis thaliana*

## Abstract

This review article deals with the pathways of cellular and global molybdate distribution in plants, especially with a full overview for the model plant *Arabidopsis thaliana*. In its oxidized state as bioavailable molybdate, molybdenum can be absorbed from the environment. Especially in higher plants, molybdenum is indispensable as part of the molybdenum cofactor (Moco), which is responsible for functionality as a prosthetic group in a variety of essential enzymes like nitrate reductase and sulfite oxidase. Therefore, plants need mechanisms for molybdate import and transport within the organism, which are accomplished via high-affinity molybdate transporter (MOT) localized in different cells and membranes. Two different MOT families were identified. Legumes like *Glycine max* or *Medicago truncatula* have an especially increased number of MOT1 family members for supplying their symbionts with molybdate for nitrogenase activity. In *Arabidopsis thaliana* especially, the complete pathway followed by molybdate through the plant is traceable. Not only the uptake from soil by MOT1.1 and its distribution to leaves, flowers, and seeds by MOT2-family members was identified, but also that inside the cell. the transport trough the cytoplasm and the vacuolar storage mechanisms depending on glutathione were described. Finally, supplying the Moco biosynthesis complex by MOT1.2 and MOT2.1 was demonstrated.

## 1. Introduction

**Figure 1:**
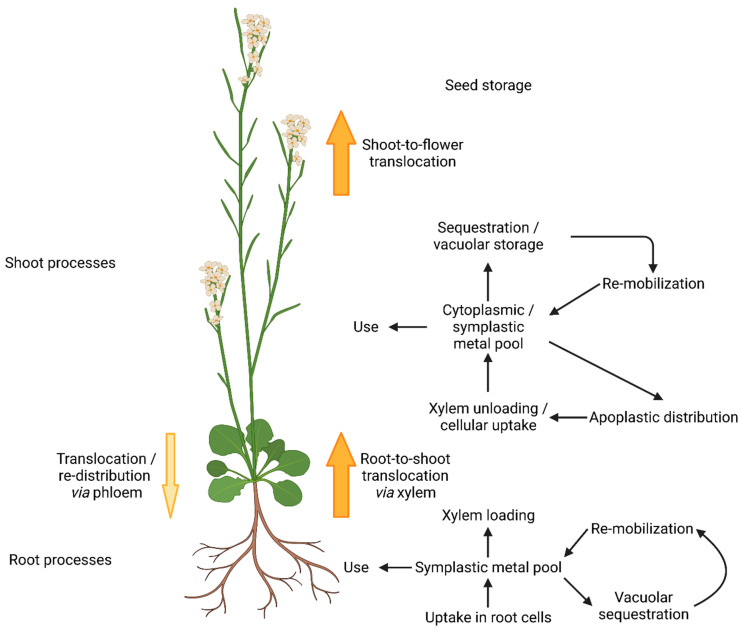
Metal homeostasis in *Arabidopsis thaliana* (according to [4]). Metal ions are taken up by specific metal transport proteins from the aqueous phase of the surrounding soil. In roots, metals can be used, stored, or translocated to the shoot via the xylem. After cellular uptake, metals form the cytoplasmic/symplastic metal pool. Apo-enzyme and prosthetic groups recruit metals from there to gain functionality. Excess metals are stored in the vacuole by sequestration. Re-mobilization makes them available for the cytoplasmic pool and they can be further distributed to the apoplast. The re-distribution to the root via the phloem plays a minor role. Translocation from the shoot to the flowers allows the storage of metals in the developing seed.

Metal homeostasis in plants is a complex and fine-tuned process, balanced between deficiency, beneficial concentration to maintain metal dependent processes, and metal toxicity symptoms [1]. A sufficient supply of metal micronutrients directly affects plant health and profound knowledge of micronutrient transport mechanisms and homeostasis can help to increase crop yields [2]. In the course of evolution, plants brought forth a variety of specialized metal transport proteins, varying in substrate specificity, expression patterns, and intra-cellular localization to meet the requirements of complex multi-cellular organisms [3]. Mechanisms not only for uptake, but also for metal distribution to organs and cells, as well as delivery to metal-requiring proteins in different cellular compartments, were developed [4] (Figure 1). Furthermore, metal storage and re-mobilization are of crucial importance [5].

The main source of metal ions for plant nutrition is the aqueous phase of the surrounding soil. Here, only low concentrations are present, making it necessary for roots to possess high-affinity transport systems for metal uptake [4]. The active transport against this gradient requires energy, either in the form of ATP hydrolysis or that of sym- or anti-port with protons or other ions [6]. ATPase hydrolyzing transporters bind a metal ion that is initially guided into a binding pocket by negatively charged residues [7] before the ATP binding forces the pocket to close and to open a pore on the opposite side of the transporter allowing the ion release. ATP hydrolysis restores the initial confirmation of the transporter [8].

After xylem loading, metal ions can be translocated to the shoot and, therefore, to leaves, flowers, or seeds [9]. The chemical properties of transition metal ions require tight control of potential binding partners inside the cell [4]. Transition metal ions show high binding affinities for electron pair donors like carboxylic, amino, and sulfide groups [10]. As a consequence, chelate complexes with low solubility can be formed, which are harmful for the cell [4]. Furthermore, transition metal ions with stronger binding affinity according to the Irving–Williams series (e.g., Cu^2+^ and Zn^2+^) can displace those with weaker binding affinity (e.g., Fe^2+^ and Mn^2+^) from their designated binding sites [10]. Consequently, a cytosolic pool of free metal ions should be avoided. Therefore, metal ions have to be transported by less affine but faster transporters and, inside the cytosol, complexed by low molecular weight compounds (LMW), like GSH, citrate, or nicotinamine, which are also involved in their vacuolar storage [11]. From there, metal ions can later be released on demand for implementation into prosthetic groups and apo-enzymes [4].

In the last years, the focus of research was laid on elucidating prominent metal metabolism pathways, especially of iron homeostasis [12], allowing one to follow the metal throughout the whole plant almost completely. However, more exotic metal micronutrients like molybdenum and the mechanisms of their plant-wide homeostasis were neglected in the last years.

## 2. The Importance of Molybdenum for Plants

Molybdenum is an essential trace element in plant nutrition [13]. The general abundance of molybdenum is thought to be 10 µg/L in most fresh-waters and <10 mg/kg in non-contaminated soils [14]. The metal does not occur in pure form in nature, but forms minerals with other elements [15]. The most common one is molybdenite (MoS_2_) due to the chalcophilic character of molybdenum [14]. The oxidation of molybdenite during weathering leads to the formation of secondary metal molybdates [15] that are bioavailable for plants [16]. Whereas alkaline soils show an increased molybdate accessibility for plants, acidic soils lead to a reduced availability [17].

**Figure 2:**
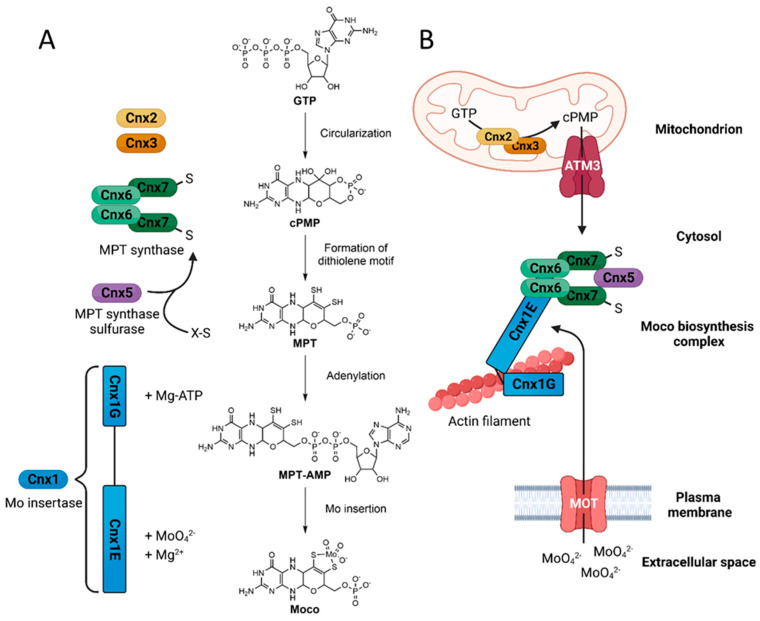
Moco biosynthesis in *Arabidopsis thaliana* [30]. Moco biosynthesis is a conserved four step pathway. (**A**) Shown are the molecular mechanism and the involved Cnx enzymes with cofactors and substrates beside the reaction step. (**B**) The cytosolic enzymes of the Moco biosynthesis undergo tight protein–protein interaction with Cnx1 acting as an anchor to the actin cytoskeleton. Complex formation benefits substrate channeling of oxygen-sensitive intermediates during biosynthesis and allows the spatial positioning near molybdate transporters (MOT).

Although organisms only depend on marginal amounts of this trace element, dysfunctions in molybdenum metabolism are lethal. The bioavailable oxyanion molybdate has to be incorporated into the unique pterin-scaffold molybdopterin (MPT) to form biologically active molybdenum cofactor (Moco) [18]. Moco is an essential prosthetic group and its four-step biosynthesis (Figure 2) is conserved in all kingdoms of life [19]. In plants, the enzymes involved are named regarding the cofactor for nitrate reductase and xanthine dehydrogenase (Cnx) nomenclature. The first step of Moco biosynthesis is the formation of cyclic pyranopterin (cPMP) from GTP catalyzed by Cnx2 and Cnx3 in the mitochondria [20]. The formed cPMP is exported by the inner mitochondria membrane ABC-transporter ATM3 into the cytosol [21]. There, cPMP is turned into molybdopterin (MPT) under the formation of a dithiolene motif catalyzed by the hetero-tetrameric MPT synthase consisting of two Cnx6 and two Cnx7 subunits. The MPT synthase has to be re-sulfurized and adenylated after each gradual transfer of a sulfur atom by the MPT synthase sulfurase Cnx5 [22]. The remaining steps of Moco biosynthesis are catalyzed by the two-domain molybdenum insertase Cnx1. The Cnx1 G-domain catalyzes the adenylation of MPT in an ATP- and Mg^2+^-dependent manner [23]. The resulting MPT-AMP is positioned in the active site of the E-domain, where the insertion of the molybdenum atom takes place with molybdate acting as substrate [24]. Molybdate is imported by specialized molybdate transporters (MOT) [25]. Moco and its intermediates are highly oxygen-sensitive; therefore, the cytosolic enzymes of Moco biosynthesis, Cnx5, Cnx6, Cnx7, and Cnx1, undergo tight protein–protein interaction resulting in the multi-enzyme Moco biosynthesis complex [26] anchored to the actin cytoskeleton by Cnx1 [27] for oxygen-free substrate channeling.

The resulting di-oxo Moco can be incorporated into the apo-enzymes of the sulfite oxidase (SO) family by direct protein–protein interaction with the molybdenum insertase Cnx1. In contrast, for the xanthine oxidase (XO) family to function, di-oxo Moco is transferred by the specialized Moco-binding proteins 2 (MoBP2) from Cnx1 to the Moco sulfurase ABA3 [28], which uses L-cysteine as donor to add a terminal sulfur to the molybdenum atom generating mono-oxo Moco [29]. The mature mono-oxo Moco is then incorporated into the user-enzymes by direct protein–protein interaction [28].

Although more than 50 molybdo-enzymes belonging to three families are known in nature, the majority are of prokaryotic origin and only a few were found in eukaryotes [31]. The families are defined by the kind of Moco. Members of the dimethyl sulfoxidase reductase family (DMSOR) are only present in bacteria and within this family Mo is bound to two dithiolene moieties from two MPT backbones [32]. In eukaryotes, only members of the SO family with di-oxo Moco and XO family (mono-oxo Moco) were identified. In plants, Moco is used by five molybdo-enzymes that catalyze key reaction steps in the metabolism of carbon, nitrogen, and sulfur. This, a broad variety of redox reaction, is catalyzed by a two-electron transfer, varying the oxidative state of the molybdenum atom between IV and VI [31]. The aldehyde oxidase (AO) and the xanthine dehydrogenase (XDH) belong to the XO family, while the nitrate reductase (NR), the sulfite oxidase (SO), and the mitochondrial amidoxime-reducing component (mARC) belong to the SO family.

AOs belong to a broad enzyme family with many members in different plants. Their broad substrate specificity suggests involvement in a plethora of metabolic processes [33]. The abscisic aldehyde oxidase AAO3 from *A. thaliana* is the most prominent member of this family. It catalyzes the last step of abscisic acid (ABA) biosynthesis [34]. The XDH is involved in purine degradation by catalyzing the reaction from hypoxanthine into xanthine and, further, the degradation to urate [35], as well as in radical oxygen species production for signaling pathways [36] and pathogen defense [37].

The mARC is involved in the detoxification of N-hydroxylated aromatic amines like N-hydroxylated nucleobases and nucleotides in mammals [38], which was also suggested for plant mARC since the reduction of a N-hydroxylated model substrate was observed [39]. While the physiological role of mARC in higher plants remains unclear, functions in the algae *Chlamydomonas reinhardtii* of detoxification of NHC N6-hydroxylaminopurine [40] as well as production of nitric oxide in a dual system with NR have been demonstrated [41]. Plant NR catalyzes the first and rate-limiting step of nitrate assimilation [42], which is of key importance for plant nutrition and underlies complex regulation mechanisms [43]. Two electrons are transferred from the reductant NADH to the prosthetic groups FAD, and passed to Haem and Moco to the substrate nitrate [33]. The plant SO is located in peroxisomes and catalyzes the reaction of sulfite to sulfate [44]. The reaction allows plants to survive during toxic SO_2_ fumigation near volcanic fumaroles [45] and forest fires [46].

A second type of molybdenum-dependent prosthetic group is the bacterial iron–sulfur molybdenum cofactor (FeMoco; [47]), exclusively imbedded in the nitrogenase, being the key enzyme for nitrogen fixation and thus important for the global nitrogen cycle [48]. Some plant families like legumes (Fabaceae) have symbiotic N_2_-fixing rhizobia living in root nodules, using the bacterial nitrogenase mechanism for plant nitrogen supplying. The molybdate uptake and supply to these nodules appears to be essential for nitrogenase activity. The produced ammonia is available to the legumes for growth and is thus indirectly also important in the nitrogen cycle of other plants, animals, and fungi as well as in sustainable food production [49].

As for other transition metals, toxic effects due to high abundance were described [4]. Mine tailings can show dramatically increased concentrations of molybdenum ranging from 100 mg/kg to 4000 mg/kg [15] with harmful potential for most plants with the exception of a few tolerant specialists [50]. Typical signs of molybdenum toxicity are yellow-orange chlorosis with brownish tints on younger leaves [14]. Whereas the effect of toxic concentrations of molybdenum is reportedly less common and is limited to those extreme examples, signs of molybdenum deficiency in crop plants are reported more frequently. In the family of *Brassicaceae*, molybdenum deficiency ranges from mottling and leaf cupping to dwarfism culminating in plant death [17].

## 3. Molybdate Transport by Specialized Membrane Transporters

The molybdate transport and homeostasis system in bacteria is quite well understood [51]. Molybdate uptake is facilitated by a high-affinity ABC-transporter family system encoded by the modABC operon in *Escherichia coli* [52]. The complex contains the proteins ModA, ModB, and ModC. While ModA binds molybdate specifically in the periplasm, ModB builds the integral membrane channel. The required energy for molybdate transport is provided by ModC which has an ATPase subunit in the cytoplasm [1]. Some bacteria have small additional (approx. 7 kDa) cytoplasmic molybdate-binding proteins that are involved in storage and homeostasis [53,54]. The modABC operon is repressed by the ModE transcription factor under low molybdenum concentrations. ModE is able to bind molybdenum and release the promotor under high molybdenum concentrations. After releasing from the modABC operon, ModE acts as an activator for the Moco biosynthesis protein [55].

*Azotobacter vinelandii* and other soil-dwelling bacteria have developed an even more advanced mechanism to enable sufficient molybdenum supply for N_2_-fixing via their FeMoco-containing enzyme nitrogenase. A tight regulation for acquisition of the cofactor-essential metals iron and molybdenum is needed [56]. Siderophores, high-affinity iron-binding compounds, are released by the bacteria into their external medium. Molybdenum will be bound in addition to iron. Excreted siderophores like protochelin, azotochelin [48], azotobactin [57], or catecholamide [58] are able to form strong complexes with molybdate, which indicates that molybdate availability is critical for nitrogenase activity. The Mo–siderophore complexes are imported through the outer membrane by specialized receptor transporters into the periplasm, where molybdate is released and sequestered by its periplasmic binding protein ModA before importing by the described ABD transporter family system [58]. Interestingly, molybdate availability upregulates azotochelin production up to 100 mM, whereas higher concentration downregulates its production [59]. Siderophores are also discussed as protecting bacteria from metal-induced oxidative stress [60].

A specialized uptake system with multiple molybdate transporters (MOTs) of the MOT1 family was developed by legumes to sufficiently supply their symbiotic N_2_-fixing rhizobia with molybdate for nitrogenase activity [61]. For example, soybean (*Glycine max*) has seven members of the MOT1 family and five members were found in *Medicago truncatula* [62]. *Mt*MOT1.2 was found to be required for molybdate delivery to the endodermis cells of the roots, while *Mt*MOT1.3 most likely introduces molybdate into nodule cells where bacterial transporters take up the delivered molybdate to build the essential nitrogenase FeMo cofactor [63]. The recently identified *Lj*MOT1 from another legume, *Lotus japonicus*, is localized in the plasma membrane and might be responsible for molybdate uptake from the soil and its distribution inside the plant rather than for Mo delivery to the nodules [64].

**Figure 3:**
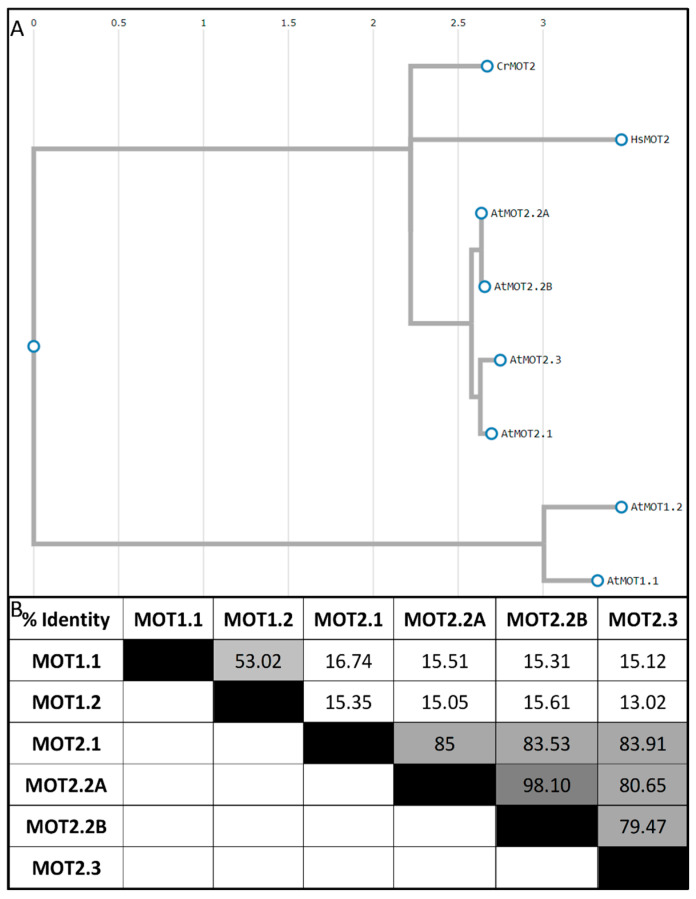
Analysis of molybdate transporter protein sequences. (**A**) Phylogenetic tree of MOT1 (AtMOT1.1 (F3N11.13), AtMOT1.2 (F5I6.6)) and MOT2 proteins (CrMOT2 (XP_001693567), HsMOT2 (BAC11137), AtMOT2.1 (NP_567786), AtMOT2.2A (NP_176646), AtMOT2.2B (NP_001077772), and AtMOT2.3 (NP_190500)). Alignment and phylogenetic reconstructions were performed using the function “build” of ETE3 3.1.2 [73] as implemented on the GenomeNet. The tree was constructed using fasttree [74]. (**B**) Identity Matrix of MOT amino acid sequences created via MUSCLE (black = 100%, darker gray = 99.9–90%, dark gray = 90–75%, gray = 75–50%, light gray = 50–20%, white = <20%).

In contrast to legumes, molybdate transporters of *A. thaliana* are well characterized. The complexity of molybdate homeostasis in a multicellular organism forced evolutions of *A. thaliana* to develop mechanisms for micro-compartmentation, safekeeping, and allocation. For all these tasks, the six identified molybdate transporters have to be highly molybdate-specific; otherwise, a precise molybdate homeostasis would be impossible [65]. These six MOTs were categorized in two independent MOT families (Figure 3) [16,66].

The MOT1 family consists of two members, MOT1.1 and MOT1.2 [65]. The MOT1 family has high similarity to sulfate transporters but are missing both the sulfate transporter motif [67] as well the STAS domain (sulfate transporter and anti-sigma factor antagonist), necessary for sulfate transport [68]. MOT1.1 is localized in the plasma membrane with the N-terminus in the apoplast and the C-terminus in the cytosol [65]. It is primarily expressed in root cells [69] and it shows high-affinity molybdate transport activity (Km of 20 nM, [16,70]). MOT1.2 is localized in the tonoplast and exports molybdate from the vacuolar storage into the cytoplasm [71]. The MOT2 family consists of three *mot2* genes coding for four MOTs, MOT2.1, MOT2.2A, MOT2.2B, and MOT2.3, which are all localized in the plasma membrane with the N-terminus in the apoplast and the C-terminus in the cytosol. The amino acid sequence of all members shows four highly conserved motifs that appear to be essential for molybdate transport activity [25]. Moreover, the members of the MOT2 family were recently found in a second pathway and were associated with a putative function as S-Adenosyl methionine (SAM) transporters located in the Golgi apparatus in *Arabidopsis* [72]. A member of the MOT2 family (*Cr*MOT2) is also found in the unicellular green algae *C. reinhardtii* [66]. It is only induced by low extracellular molybdate concentration, which suggests a role in maintaining intracellular molybdate concentrations, while *Cr*MOT1 is the main importer [66]. MOT2 orthologues were described in most eukaryotes, including plants and animals. Yeast cells transformed with MOT2 from both *Chlamydomonas reinhardtii* and Homo sapiens (*Hs*MOT2) showed specific molybdate uptake activity (Km = ~550 nM) which is still in the range of high-affinity systems [66]. Also, molybdate transport activity for all members of the MOT2 family from *A. thaliana* was shown using a yeast-based growth inhibition system on chlorate [25]. Furthermore, all MOTs were ranked regarding their transport activity in the presence of 100 nM molybdate: MOT1.1 and MOT1.2 showed the highest transport activity, followed by MOT2.3, MOT2.2B, MOT2.2A, and lastly MOT2.1.

## 4. Following Molybdate along Its Way through *A. thaliana*

**Figure 4:**
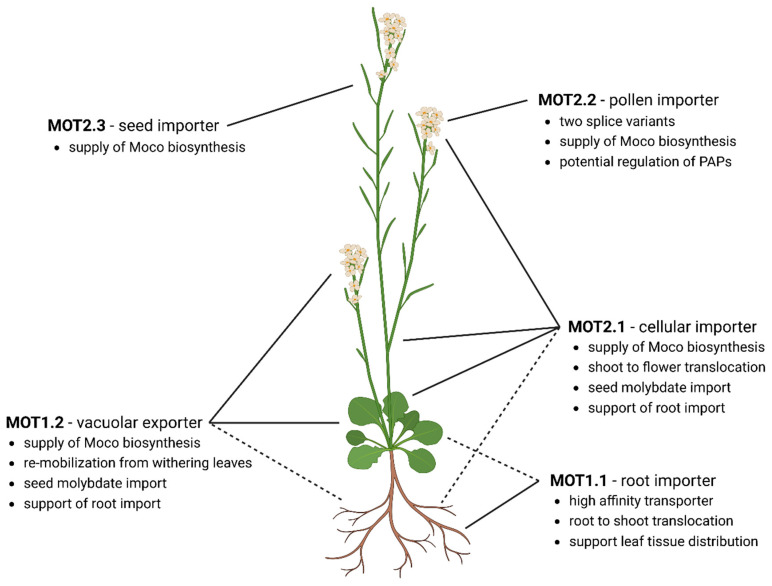
Organ-specific localization and functions of molybdate transporters for homeostasis in *A. thaliana*. MOT1.1. is the main root importer and involved in root-to-shoot translocation. MOT2.1 is the main molybdate distributor and cellular importer. MOT2.1 and MOT1.2 deliver the Moco biosynthesis. Stored molybdate will be released by MOT1.2 while senescent to re-mobilize molybdate from the vacuole. In the flower, MOT1.2 and MOT2.1 maintain the cellular molybdate homeostasis. Additionally, MOT2.3 supports the supply of Moco biosynthesis due to an increased amount of molybdo-enzyme activity during seed development. MOT2.2 is exclusively produced in the pollen. Here, two splice-variants might fulfil different tasks. Whereas MOT2.2A interacts with the Moco biosynthesis, MOT2.2B shows no interaction and supplies molybdate for regulatory purposes of purple acid phosphatases. Dashed lines indicate supportive MOT roles.

In general, molybdate is a scarce soil component [14] with a concentration of 10 nM [16]. However, the concentration of absorbing iron oxides, certain organic compounds, and soil acidity can decrease the phyto-availability of molybdate [17]. Furthermore, uptake of molybdate from soil into the plant is directed against a concentration gradient and, thus, requires energy [6]. These requirements are perfectly met by MOT1.1 (Figure 4), due to its production in the plant root [69], its presence in the plasma membrane [65], and a striking high-affinity molybdate transport activity [16]. Analyses of a loss-of-function *mot1.1*-KO grown under limited molybdate availability in a hydroponic growth system revealed a retarded growth behavior and signs of molybdate deprivation like a pale leaf color and necrotic areas [65]. The same KO line showed decreased molybdate concentrations in root and shoot [16] caused by a strongly reduced molybdate uptake ability and a resulting dramatic decrease in nitrate reductase activity [65]. A lack of interaction of MOT1.1 with the molybdenum insertase Cnx1 underlines that it is solely involved in the root molybdate import and does not participate in supplying molybdate for the Moco biosynthesis complex.

MOT1.1 involvement in root-to-shoot translocation and xylem unloading into leaf tissue is assumed due to reduced molybdate concentrations in shoots of the *mot1.1-*KO [16,69] and the strong *mot1.1* expression in leaf venation [65,69]. A supportive role in molybdate root-to-shoot translocation after its uptake by MOT1.1 is hypothesized for MOT2.1 as it is produced in the vascular tissue of the roots and shoot [25]. After xylem unloading into leaf tissue, molybdate finds itself in the apoplast and needs to be transported across the plasma membrane into the cytosol by a cellular importer. A study has shown that the plasma membrane transporter MOT2.1 realizes this import, due to its equal production in the tissue of young leaves and the induction of *mot2.1* by the presence of the substrate molybdate. Furthermore, a negatively affected NR activity in absence of MOT2.1 while molybdate uptake is unaffected supports its importance for cellular import [25].

**Figure 5:**
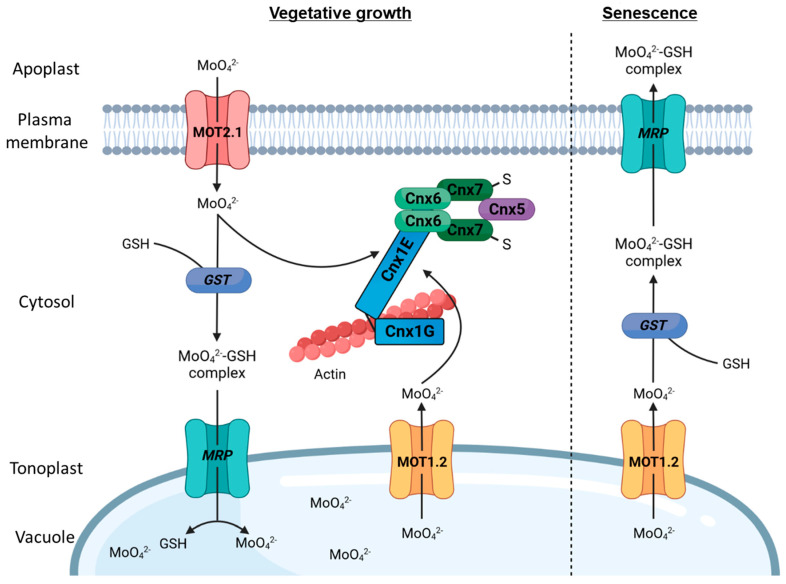
Cellular molybdate transport in *Arabidopsis thaliana*. MOT2.1 is the main cellular importer and delivers molybdate to the Moco biosynthesis. Excess molybdate is complexed by GSH, likely by glutathione S-transferase (GST), and transported into the vacuole. It releases on demand by the tonoplast-localized MOT1.2 in direct interaction with the Moco biosynthesis or during senescence for export via the GSH–molybdate complex.

Inside the cell (Figure 5), prevention of a potential harmful pool of free heavy metal ions is aimed for. To do so, MOT2.1 hands molybdate directly over to molybdenum insertase Cnx1 for direct supply to the Moco biosynthesis. This substrate channeling guarantees efficient reaction rates of Moco biosynthesis and, in parallel, avoids formation of a free pool of hazardous molybdate ions [25]. Studies show that a surplus of imported molybdate is stored in the vacuole [71] without free diffusion inside the cytosol. Until now, no vacuolar importer has been found. However, it might be imported in a complexed form as the formation of a molybdate–glutathione complex via the carboxylation of glycine and the thiol in cysteine was shown under in vitro conditions [25]. Glutathione (GSH) is a cellular LMW compound and a part of the detoxification strategy of heavy metal ions in plants [75]. In general, a formed metal–GSH complex is tagged for vacuolar storage [76] and is transported into the vacuole in disregard of the incorporated heavy metal ion by members of the multidrug-resistance-associated protein (MRP) family [77]. After dissociation due to the pH shift [78], the heavy metal ion is non-specifically sequestered by organic acids or free amino acids present in the vacuole and rendered harmless [78]. The molecular mechanisms of the complexation in cytosol and the molybdate–GSH complex import into the vacuole remain unclear, but the usage of glutathione S-transferases as well as that of unspecific metal–GSH import was hypothesized [25].

As molybdate is a valuable micronutrient, vacuolar storage is supposed to be of a temporary character, implying the presence of an exporter from the storage organelle. Here, MOT1.2 comes into play functioning as tonoplast exporter [71]. MOT1.2 is expressed globally in leaf tissue with observed upregulation in older senescent leaves [65]. Interestingly, loss-of-function *mot1.2*-KO accumulates molybdate in the vacuole, resulting in increased molybdate amounts of rosette leaves [71]; an effect on vegetative growth and NR activity, however, could not be observed [65]. As shown for MOT2.1, interaction of MOT1.2 with Cnx1 was demonstrated, implying a physiological role as vacuolar molybdate exporter and, next to MOT2.1, as an additional molybdate supplier for the Moco biosynthesis [65]. This interplay between cellular import and vacuolar export guarantees a sufficient molybdate supply for Moco biosynthesis in all growing stages.

Macro- and micronutrients like molybdate have to be re-mobilized from leaves to seeds during senescence to be given to the next generation [9]. In senescent leaves, the transcription level of the vascular exporter MOT1.2 is increased and loss of MOT1.2 leads to a highly reduced level of molybdate in the seeds, which shows the importance of MOT1.2 for molybdate relocation and conservation during senescence [71]. It can be assumed that molybdate–GSH complex formation is also mediated after vacuolar export. It was observed that several metal–LMW complexes are cleaved before cellular export and then loaded to the xylem stream individually [10]. However, an export through the plasma membrane of intact metal–LMW complexes is also described [79]. To prevent a direct re-import of molybdate, production of MOT2.1 in senescent leaf tissue is decreased completely, while being concentrated in the main leaf venation for loading molybdate to the xylem [25]. After export, molybdate needs to be translocated from leaves to flowers via the xylem of the shoot. MOT2.1 is distinctly produced in this tissue, designating MOT2.1 as main molybdate distributor in *A. thaliana* [25].

The last step of molybdenum allocation is its import into flower cells and developing seeds. Here, both MOT2.2 and MOT2.3 are exclusively expressed in pollen and ovaries, respectively (Figure 4). Therefore, their role during sexual reproduction is implied [25]. Beside these specialized MOTs, the universal distributor MOT2.1 is also broadly produced in flowers and their connected shoot [25]. Furthermore, MOT1.2 was also found strictly concentrated in fertilized ovaries [65]. The fact that four MOTs in total are produced in flowers suggests their importance and a complex network of transport processes.

MOT2.2 is exclusively produced in pollen and the corresponding gene encodes two splice variants: the full-length MOT2.2A and MOT2.2B lacking the first 41 amino acids [25]. Whereas both proteins are localized in the plasma membrane, only MOT2.2A shows interaction with the Moco biosynthesis via Cnx1 indicating different roles for these splice variants [25]. In maize, a hint for this additional and specialized supply via MOT2.2 with molybdate was found: the development of smaller anthers and stamen, as well as shriveled pollen with poor germination rates, was associated with molybdate deficiency in reproductive tissue [17]. Therefore, the need for an additional MOT might have developed during evolution for sufficient molybdate content in *A. thaliana*. The necessity of this special splice variant MOT2.2B without any Moco biosynthesis complex interaction might be an involvement of molybdate in phosphate metabolism [25]. In a recent study, molybdate acted as the most prominent purple acid phosphatases inhibitor, which abolishes the enzymatic activity of the *At*PAP15 almost completely at a molybdate concentration of 250 µM. *Atpap15* can hydrolyze phytic acid, the main storage form of phosphor in plant seeds and pollen grains [80]. A premature release of phosphate in pollen or seeds can lead to early pollen germination or unfavorable metabolic processes during pollen maturation. Thus, it is clear that the presence of molybdate in pollen is of crucial importance. In contrast, MOT2.2A is directly interacting with Moco biosynthesis. Moco is important for producing of the phytohormone abscisic acid (ABA) via the molybdo-enzyme abscisic aldehyde oxidase [34]. ABA is involved in pollen germination and pollen tube growth [81]. Taken together, this demonstrates the importance of two MOT2.2 splice variants with different roles.

ABA is also the key hormone that promotes seed dormancy during seed development and supports post-germination growth [82]. ABA originates from both zygotic tissue and maternal tissue. Even though the majority originates from vegetative tissue and is transported to the seed, ABA production in seed coating tissues might also contribute [83]. In conclusion, high Moco demands in the ovaries due to an increased activity of molybdo-enzymes requires an additional mechanism for molybdate supply in this tissue. The ovary-expressed transporters MOT2.3 (plasma membrane protein) and MOT1.2 (tonoplast) interact with Cnx1 to fulfill the role in supplying the Moco biosynthesis [25]. In addition, the generally expressed MOT2.1 might support this function due to an increased Moco demand in this tissue. However, expression of two importers in ovaries supports fast rescue of all available molybdate for the next generation [25].

## 5. Outlook

Within this review, molybdate’s pathway through the plant, within the cell and up to its usage for Moco biosynthesis, was followed for *Arabidopsis thaliana*. To accomplish all specific tasks in a complex organism with different organs and life cycles, various transporters and mechanisms are necessary. The high degree of conservation of Moco biosynthesis and the dependence of all plants on molybdate, especially for nitrogen fixation, suggests that these mechanisms are present universally in all plant species.

Several molybdate transporters have already been found in other herbaceous plants, but nothing is known regarding how molybdate homeostasis is regulated in multi-annual plants like trees, which have a completely different life cycle. Therefore, molybdate pathways not possible to investigate in *A. thaliana* are the storage in wood or buds over winter and its reactivation in spring. The mechanisms and the involved MOTs required for these pathways are still unknown. In this context, it should also be investigated whether complexation of molybdate with GSH or other molecules during xylem transport protects the plant from undesirable heavy metal reactions. Another unanswered question is whether glutathione S-transferases are involved in the complexation and which transporters play a role in the import into the vacuole.

Since the different molybdate transporters have their own very specific tasks, further research should enlighten whether these functions can be recognized within the protein structure. The aim will be to investigate the transporter’s molybdate binding site and why some interact with Cnx1 while others do not. In this context, the structural function of the sequence segment in MOT2.2, which distinguishes the two splice variants from each other, should also be clarified.

When regarding plants in their natural environment, the question arises whether certain environmental conditions can affect the uptake and transport of molybdate within the plant. For example, it is known that acidic soils can lower molybdate availability. In contrast, feedback regulation under molybdate excess would be interesting: how do plants react to high molybdate concentrations in the soil, and are there protection mechanisms to prevent excessive uptake of toxic heavy metals?

Finally, an application-oriented perspective is of interest. A future research topic should deal with possible benefits of the gained knowledge for agriculture: are there problems with molybdate availability in acidic or mineral-poor soils, and can its uptake in field crops be increased by producing crops with optimized molybdate transport capacity? The knowledge gained in the studies presented could therefore have direct benefits for efficient and sustainable agriculture.

## Data Availability

No new data were created for this article.
